# Regulation of gap junction channels and hemichannels by phosphorylation and redox changes: a revision

**DOI:** 10.1186/s12860-016-0099-3

**Published:** 2016-05-24

**Authors:** Kristin Pogoda, Petra Kameritsch, Mauricio A. Retamal, José L. Vega

**Affiliations:** Walter Brendel Centre of Experimental Medicine, Ludwig-Maximilians-Universität München and Munich University Hospital, München, Germany; DZHK (German Centre for Cardiovascular Research), partner site Munich Heart Alliance, München, Germany; Centro de Fisiología Celular e Integrativa, Facultad de Medicina, Clínica Alemana Universidad del Desarrollo, Santiago, Chile; Experimental Physiology Laboratory (EPhyL), Antofagasta Institute, Universidad de Antofagasta, Antofagasta, Chile

**Keywords:** Gap junction, Channels, Hemichannel, Phosphorylation, Nitric Oxide, Redox

## Abstract

Post-translational modifications of connexins play an important role in the regulation of gap junction and hemichannel permeability. The prerequisite for the formation of functional gap junction channels is the assembly of connexin proteins into hemichannels and their insertion into the membrane. Hemichannels can affect cellular processes by enabling the passage of signaling molecules between the intracellular and extracellular space. For the intercellular communication hemichannels from one cell have to dock to its counterparts on the opposing membrane of an adjacent cell to allow the transmission of signals via gap junctions from one cell to the other. The controlled opening of hemichannels and gating properties of complete gap junctions can be regulated via post-translational modifications of connexins. Not only channel gating, but also connexin trafficking and assembly into hemichannels can be affected by post-translational changes. Recent investigations have shown that connexins can be modified by phosphorylation/dephosphorylation, redox-related changes including effects of nitric oxide (NO), hydrogen sulfide (H_2_S) or carbon monoxide (CO), acetylation, methylation or ubiquitination. Most of the connexin isoforms are known to be phosphorylated, e.g. Cx43, one of the most studied connexin at all, has 21 reported phosphorylation sites. In this review, we provide an overview about the current knowledge and relevant research of responsible kinases, connexin phosphorylation sites and reported effects on gap junction and hemichannel regulation. Regarding the effects of oxidants we discuss the role of NO in different cell types and tissues and recent studies about modifications of connexins by CO and H_2_S.

## Background

Connexins (Cxs), a protein family with 21 members in humans, share some important functional and structural characteristics. Thus, Cxs are catalogued as transmembrane proteins, containing four transmembranous domains, two extracellular loops, one cytoplasmic loop and both cytoplasmic N- and C-terminal domains facing the cytoplasm. To constitute a gap junction (GJ) channel, six Cx subunits first assemble into a hemichannel (HC), also known as connexon that docks to another HC located in a neighbouring cell to finally form an intercellular GJ channel [1]. GJs allow the diffusion of ions, second messengers and small molecules up to ~1.8 kDa between adjacent cells [2, 3], whereas HCs allow the exchange of small molecules between the cytoplasm and the extracellular space [4–6]. The life cycle of Cxs, their cellular transport, GJ assembly, stability, degradation, the channel gating and selectivity properties are regulated via post-translational modifications and interactions with other cellular proteins [7–9]. Because phosphorylation and oxidation/reduction associated processes are found widely in all tissues, we will summarize and discuss the current knowledge of these two mechanisms on the regulation of GJs and HCs.

## Regulation of GJs and HCs by phosphorylation

Cxs have multiple phosphorylation sites, e.g. 21 of these sites have been described for Cx43 [7, 8]. The phosphorylation of Cxs occurs primarily at the C-terminal region, but Cx36 and Cx56 can also be phosphorylated within the cytoplasmic loop [10, 11]. The very short Cx26 has a C-terminal region with only 11 amino acids and was believed to be a non-phospho-protein since a phosphorylation in this protein was not detectable in hepatocytes [12]. Many years later Locke et al*.* found a possible phosphorylation site at the Cx26 N-terminal region using HeLa cells as exogenous expression system, but phosphorylation of Cx26 in cells that endogenously express this protein, like in liver cells, has not be detected yet [13, 14]. The formation of functional GJs does not seem to require Cx phosphorylation since truncated Cx43 lacking most of the cytoplasmic C-terminal region were still able to form functional GJs, albeit with different conductance than those formed by wild-type Cx43 [15, 16].

Post-translational phosphorylation of Cxs can regulate channel properties of both HCs and GJs [17]. Additionally, Cx phosphorylation also correlates with changes of Cx trafficking, GJ assembly and stability [8, 18–20]. Phosphorylation of Cxs occurs at different stages of the Cx life cycle [21]. As typical integral membrane proteins, Cxs are synthesized in the endoplasmic reticulum (ER), and depending of the Cx type they can pass or not through the Golgi network, form HCs and move to the plasma membrane where they build GJ structures [2, 22]. By far the most extensively studied Cx is Cx43, which is widely and predominantly expressed in mammalian cells [7, 8].

### Regulation of GJs by phosphorylation

The half-life of Cx43 has been reported to be about 5 h in liver hepatocytes and 1.3 h in the heart [19, 23]. Cx43 phosphorylation by protein kinase p34cdc2 has been linked to GJ internalization at the onset of mitosis [19]. Turnover, internalization and degradation are highly associated with phosphorylation/dephosphorylation events and can be triggered by a variety of stimuli (e.g., growth factors, extracellular matrix interactions, ischemia, wounding, inflammation, etc.) [9, 19]. These phosphorylation changes are related with changes in the gap junction intercellular communication (GJIC) and may be necessary for normal cell cycling [19]. Therefore, this GJ turnover is apparently a response to environmental conditions with increasing or decreasing GJIC rates [24].

Gating kinetics of GJ channels can be modulated in a rapid and reversible manner by direct phosphorylation of Cxs, which changes the extent of GJIC. GJs can thereby undergo conformational changes in order to modify channel gating as described in the “ball and chain”, “cork gating” or “particle-receptor” models [25]. The phosphorylation enables the C-terminal region to interact with either the pore-forming region or an intermediary molecule to form a complex that results in channel closure [25].

**Fig. 1:**
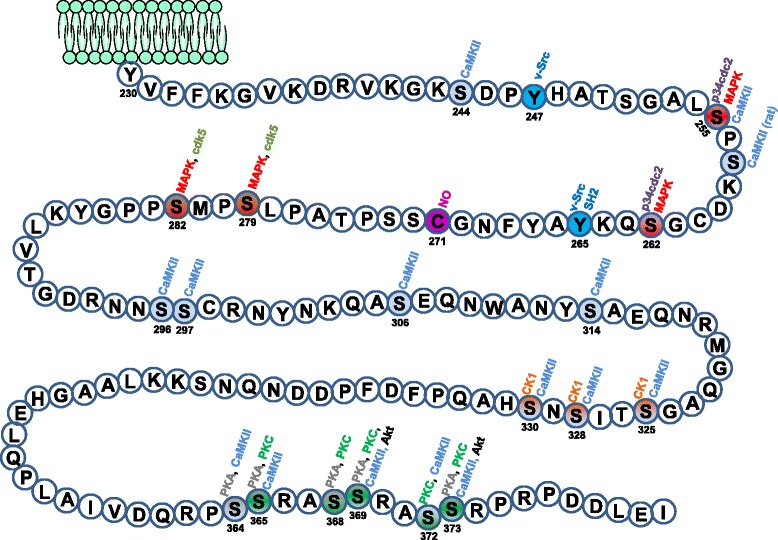
Post-translational modified sites of Cx43

In general, the phosphorylation of Cxs can occur through serine/threonine kinases or tyrosine kinases. Among the serine/threonine kinases that phosphorylate Cxs are e.g. protein kinase C (PKC), MAP kinase (MAPK), cAMP-dependent protein kinase A (PKA), casein kinase (CK), p34cdc2, protein kinase G (PKG), Ca^2+^/calmodulin-dependent kinase II (CaMKII), but also the tyrosine kinase Src is able to phosphorylate Cxs [7, 18]. Some residues may be targets for multiple protein kinases implying varying influences on GJs under different physiological or pathophysiological conditions [26]. Reported phosphorylation sites of connexins, responsible kinases and the effect on GJs are summarized in Table 1 and for Cx43 shown in Fig. 1.

**Table 1:** Reported phosphorylation sites of connexins, responsible kinases and the effect on gap junctions

Kinases	Connexins	Phosphorylated residues	Reported effects on GJs	References
PKA	Cx32	Ser233	increased GJIC	[18, 36, 37]
	Cx35/36	Ser110, Ser276	reduced GJIC	[38–41]
			increased GJIC	
	Cx40	n/a	increased GJIC	[42, 43]
	Cx43	serines 364, 365, 368, 369, 373	rapid GJ assembly, increased GJIC	[7, 27–34]
	Cx50	Ser395	increased GJIC	[35]
AKT (PKB)	Cx43	Ser373	increased GJ size and GJIC	[44]
	Cx50	n/a	increased GJIC	[45]
PKC	Cx43	serines 365, 368, 369, 372, 373	decreased GJ assembly, decreased GJIC, reduced half-life of Cx43, in ischemic hearts decreased coupling by Ser368 phosphorylation (56) or de-phosphorylation (55)	[7, 18, 46–56]
	Cx32	Ser233	n/a	[18, 37, 57]
	Cx56 chicken homologue of Cx46	Ser118	decreased GJIC	[11, 18, 58]
PKG	Cx35/36	Ser110, Ser276, Ser289	decreased GJIC	[59]
CaMKII	Cx43	serines 244, 255, 257, 296, 297, 306, 314, 325, 328, 330, 364, 365, 369, 372, 373	de-phosphorylation of Ser306 during ischemia reduced GJIC (62)	[60–63]
	Cx32	n/a	n/a	[37]
	Cx36	n/a	increased GJIC	[64–66]
	Cx45	Ser326, Thr337, serines 381, 382, 384, 385, 387, 393	n/a	[67]
CK1	Cx43	serines 325, 328, 330	increased GJ assembly	[26]
	Cx45	serines 326, 382, 384, 387, 393	n/a	[67]
Cdk5	Cx43	Ser279, Ser282	phosphorylation prevents membrane targeting, promotes proteasome dependent degradation	[69]
P34cdc2	Cx43	Ser255, Ser262	GJ internalization	[70, 71]
MAPK	Cx43	serines 255, 262, 279, 282	GJ internalization, decreased GJIC	[54, 70, 72–75]
	Cx50	n/a	n/a	[76]
Src	Cx43	Tyr 247, 265	reduced GJIC	[77–79]
EGFR tyrosine kinase	Cx32	Tyr 243	n/a	[82]

n/a, not applicable

### Phosphorylation of connexins by serine/threonine kinases and its effect on GJs

#### cAMP-dependent protein kinase A (PKA)

In a variety of cell populations, an increase of cAMP concentration is correlated with an upregulation of GJIC, but there are also reports showing that cAMP increases had an inhibitory effect or no effects upon GJIC [27]. For Cx43, it has been demonstrated that elevated intracellular levels of cAMP increased GJIC in different systems [28–31]. Paulson et al*.* have shown that elevated levels of cAMP enhanced the membrane trafficking of Cx43 and could trigger a rapid GJ assembly at the cell surface [27]. The increased GJ assembly involved the activation of PKA and phosphorylation of Cx43 at Ser364 as shown in a study of TenBroek et al*.* [32]. The treatment of primary granulosa cells with follicle-stimulating hormone (FSH), which increases the intracellular level of cAMP, induced the phosphorylation of Cx43 at Ser365, Ser368, Ser369 and Ser373 [33]. It is therefore conceivable that these phosphorylation events occur in a PKA dependent way [33]. While Ser364 is phosphorylated by PKA [32], Ser365 can be phosphorylated either by PKA or PKC [7]. Shah et al*.* have found that Cx43 is a relatively poor substrate of PKA compared to PKC, but the phosphorylation at Ser364 by PKA seems to be important for subsequent phosphorylation of Cx43 by PKC [34]. In addition, also other Cx types are phosphorylated by PKA. Thus, in vivo phosphorylation of Cx50 (Ser395) by PKA in chicken lens has been shown to promote dye permeability of Cx50 GJs and appears therefore to be important for metabolic coupling and transport in lens fibers [35]. The PKA dependent in vitro phosphorylation of Cx32 (at Ser233) in hepatocytes was associated with an increase in GJIC [18, 36, 37].

A negative regulation by PKA was reported for GJs formed by Cx35. In HeLa cells expressing Cx35, the application of a PKA activator reduced GJIC, while a PKA inhibitor increased GJIC [38]. In vitro phosphorylation studies showed that PKA phosphorylates Cx35 at Ser110 (in the intracellular loop) and Ser276 (in the carboxyl terminus) [38, 39]. A study from Li and colleagues showed that PKA phosphorylated Cx35 directly in photoreceptors and increased GJIC [40]. The explanation for these apparently contradictory findings of PKA to reduce Cx35-GJIC comes from a study of Kothmann et al*.* They demonstrated that the dopamine induced uncoupling of AII amacrine cells resulted from PKA activation of protein phosphatase 2A (PPA2) and subsequent dephosphorylation of Cx35 [41]. These results suggest that the effect of PKA activity on Cx35 GJIC can be positively affected by PKA dependent phosphorylation of Cx35 and negatively regulated by PKA dependent activation of PPA2 and subsequent dephosphorylation of Cx35.

A role for PKA in the regulation of GJIC was also reported for Cx40. Administration of cAMP resulted in a mobility shift of Cx40 on western blot and increased the permeability of GJs formed by Cx40 in transfected SKHep1 cells [42]. Moreover, a study of Bolon et al*.* demonstrated that lipopolysaccharide (LPS) and hypoxia/reoxygenation (H/R) reduced GJIC of wild-type microvascular endothelial cells but not in Cx40 null cells, and this was associated with a decreased PKA specific phosphorylation of Cx40 [43].

#### AKT (PKB)

AKT dependent phosphorylation of Cx43 at Ser373 increased GJ size and communication by preventing the binding of ZO-1 to Cx43 [44]. Phosphorylation of Ser373 was mainly observed in larger gap junctional structures at the membrane and was increased in epithelial cells and skin or heart tissue after wounding or hypoxia. This phosphorylation site might therefore act as a molecular switch to increase GJIC under these conditions [44]. A potential regulation of Cx50 but not Cx46 by PI3K signaling has been described in a study of Martínez and colleagues [45]. They demonstrated that PI3K/AKT activation upregulated Cx50 GJ conductance whereas inhibition of PI3K or AKT decreased Cx50 mediated GJ conductance [45].

#### Protein kinase C (PKC)

A PKC dependent phosphorylation of Cxs has been shown to affect GJIC in a negative manner decreasing GJ conductance or reducing dye coupling [18, 46]. The phosphorylation of Cx43 by PKC has been demonstrated by different groups and involves the amino acids Ser365, 368, Ser369, Ser372 and Ser373 [7, 46–49]. It has been shown in many different cell types that the activation of PKC increased the phosphorylation of Cx43, which was associated with decreased GJ assembly, downregulation of GJIC and a reduced half-life of Cx43 [18, 50]. A correlation between PKC dependent Ser368 phosphorylation of Cx43 and decreased GJIC has been reported by several groups [47, 48, 51–53]. For example, Liao et al*.* showed that Ser368 phosphorylation by PKC was accompanied by a loss of Cx43 from GJs, an increased ubiquitination of Cx43 and reduced GJIC [51]. This was confirmed by Wang et al*.*, who found an increased localization of Cx43 in the membrane and enhanced GJIC when PKC mediated Ser368 phosphorylation of Cx43 was inhibited [52, 53]. The effect of the tumor-promoting phorbol ester 12-O-tetradecanoylphorbol 13-acetate (TPA) on Cx43 GJs was reported by Sirnes and colleagues [54]. They demonstrated that the TPA induced inhibition of GJIC was dependent on Cx43 phosphorylation not only by PKC on Ser368, but also by MAPKs on Ser255 and Ser262 [54].

The role of Ser368 phosphorylation of Cx43 on the electrical conductance during myocardial ischemia does not appear to be sufficiently clear. A study by Axelsen and colleagues demonstrated that Ser368 became dephosphorylated during ischemia in isolated rat hearts and that was associated with a similar decrease in electrical coupling [55]. A preservation of Ser368 phosphorylation should therefore contribute to an improved electrical coupling during ischemia [55]. However, Ek-Vitorin and colleagues found a reduced GJ conductance with increased phosphorylation of Ser368 in Cx43 during ischemia in excised mice hearts [56]. Further research may clarify the role of Ser368 phosphorylation and will explain these contradictory findings.

The phosphorylation of Cx32 at Ser233 in a PKC dependent manner has been described in freshly isolated hepatocytes [18, 37, 57]. This amino acid can additionally be phosphorylated by PKA [37]. In chicken lens cultures expressing Cx56 (the chicken homologue of Cx46) the treatment with TPA resulted in an increased phosphorylation at Ser118 of the intracellular loop of Cx56 in a PKC dependent manner [11]. Moreover, the TPA-dependent phosphorylation of Ser118 decreased GJIC and accelerated degradation of Cx56 [11, 18, 58].

#### Protein kinase G (PKG)

Cx35 (the fish ortholog of mammalian Cx36), that is expressed in neurons, can be phosphorylated by PKG [59]. In vitro studies with stably transfected HeLa cells identified three PKG phosphorylation sites in Cx35: Ser110 (within the intracellular loop), Ser276 and Ser289 (within the C-terminal tail). The PKG dependent phosphorylation of Cx35 was regulated by NO that can induce the activation of soluble guanylyl cyclase/cGMP/PKG [59]. Both, application of NO donors or activation of PKG in the absence of exogenous NO caused Cx35-mediated uncoupling in HeLa cells [59].

#### Ca^2+^/calmodulin kinase II (CaMKII)

Ca^2+^/calmodulin kinase II plays a critical role in the regulation of Ca^2+^ homeostasis, transcription and apoptosis, as well as in ischemic heart diseases. In cardiac diseases, such as heart failure or myocardial ischemia and infarction, an increased expression or activation of CaMKII has been shown [60]. Calmodulin (CaM) can modulate GJ gating properties by activating CaMKII or by direct interaction with the Cx proteins mediating Ca^2+^-induced uncoupling of GJs. The GJ inhibition by Ca^2+^-CaM can be prevented by CaM blockers or CaM antagonists [61]. A co-localization of CaMKII with Cx43 has been found at the border zone of infarcted hearts [62]. Huang and colleagues identified 15 serine phosphorylation sites for CaMKII in the carboxyl terminus of Cx43. They investigated the phosphorylation of Cx43 by CaMKII in vitro using fusion proteins containing the C-terminal region of Cx43. The serine residues 244, 255, 257, 296, 297, 306, 314, 325, 328, 330, 364, 365, 369, 372 and 373 were identified by mass spectrometry as putative in vitro phosphorylation targets of CaMKII [60]. Dephosphorylation of Ser306 has been shown during ischemia in rat hearts and contributes to a reduced coupling [63]. Phosphorylation of Cx43 at Ser325/328/330 was previously shown by Cooper and Lampe, but in a CK1 mediated manner [26]. Cx32 can also be phosphorylated by CaMKII in addition to PKA and PKC, as demonstrated by Sáez et al*.,* in purified liver GJs and in hepatocytes [37]. An interaction of Cx36 with CaMKII and its phosphorylation by CaMKII was demonstrated by Alev and colleagues [64]. They identified two binding sites of CaMKII at the cytoplasmic loop and close to the carboxyl-terminal region of Cx36 [64]. An increased GJ conductance has been shown to correlate with increased intracellular Ca^2+^ levels that induced the activation of CaMKII. Therefore, the enhancement of the GJ conductance could be mediated by a phosphorylation of Cx36 by CaMKII [65, 66]. Also Cx45 can be phosphorylated by CaMKII as well as by CK1 as demonstrated by Bao and co-workers. They identified eight Ser/Thr residues (Ser326, Thr337, serines 381, 382, 384, 385, 387 and 393), that were phosphorylated by CaMKII by mass spectrometry [67]. Some of these residues are also targets of CK1 (Ser326, 382, 384, 387 and 393) [67]. It remains to be elucidated, whether these residues are indeed phosphorylated in vivo and how they may affect GJIC and/or GJ assembly [67].

#### Casein kinase (CK)

Phosphorylation of Cx43 by CK1 at Ser325, Ser328 and Ser330 has been reported to promote GJ assembly [26]. As mentioned above, CK1 can also phosphorylate Cx45 at Ser326, 382, 384, 387 and 393, as shown by Bao et al*.* [67]. On the other hand, Yin et al*.* reported that Cx45.6 (the avian ortholog of mammalian Cx50) is phosphorylated by CK2 in vitro at Ser363, but not by CK1 and they also observed that this phosphorylation event led to destabilization and degradation of Cx45.6 [68].

#### Cyclin-dependent kinase 5 (Cdk5)

Cdk5 is involved in developmental processes of the brain. Cdk5 directly phosphorylates Cx43 at Ser279 and Ser282 and thereby preventing the membrane targeting of Cx43 and promoting its proteasome dependent degradation [69]. Cx43 expression in neurons is involved in regulating neuronal progenitors migration and positioning in the developing brain by controlling the interaction of astrocytes with neurons [69]. Therefore, the membrane localization and degradation of Cx43 in neurons during neuronal differentiation appears to be regulated by Cdk5 dependent phosphorylation [69].

#### Cyclin dependent kinase p34cdc2

Phosphorylation of Cxs during cell cycle progression can be regulated by p34cdc2 playing an important role for the initiation of mitosis [70]. The reduction of GJIC during mitosis is accompanied by increased phosphorylation of Cx43 at Ser255 and Ser262 by p34cdc kinase activity. This cell cycle regulated phosphorylation of Cx43 is associated with cells rounding and GJ internalization [70, 71].

#### MAPK

Serine residues 255, 262, 279/282 of Cx43 are target sites for MAPK phosphorylation [54, 72–74]. In endothelial cells, phosphorylation of Cx43 at Ser255, 262 and 279/282 by MAPK ERK1/2 in response to stimulation with VEGF has been found [72]. This was associated with a rapid GJ internalization and correlated with an inhibition of GJIC, which was restored within 1–2 h after VEGF treatment [72]. Also treatment with phorbol esters (TPA), lysophosphatic acid (LPA) or EGF and subsequent activation of the EGF receptor induced a phosphorylation of Ser255, Ser262, Ser278/282 of Cx43 leading to a rapid disruption of GJIC [54, 73, 74]. Accordingly, mutations of Ser279 and 282 restored GJ assembly [75]. Ser255 has been reported to be phosphorylated by both MAPK and p34cdc2 [70]. An increased GJ coupling induced by activation of the MAPK ERK1/2 signaling has been demonstrated in paired *Xenopus* oocytes and primary lens epithelial cells [76]. In these cells the enhanced coupling was mediated by expression of Cx50, but not Cx46. However, it remains unclear whether Cx50 becomes directly phosphorylated by MAPKs or indirectly via other kinases activated by the MAPK pathway [76].

### Phosphorylation of Cxs by tyrosine kinases and its effect on GJIC

Activation of the oncogene and tyrosine kinase Src leads to acute down-regulation of GJIC [77]. Src can affect GJs in different ways, as shown for Cx43 either by a direct tyrosine phosphorylation or by activating downstream effector kinase pathways [78]. Lin and colleagues proposed a model for the interaction of Cx43 with Src and its phosphorylation by Src leading to the disruption of the GJIC [79]. At first, the SH3 domain of Src binds to the proline rich region of Cx43 (Pro274-Pro282) that brings the kinase domain of Src close to Tyr265. The phosphorylated Tyr265 facilitates the binding of the SH2 domain of Src that stabilizes the interaction of Cx43 and Src. The enhanced interaction induces the phosphorylation of Tyr247, which triggers the closure of GJs built by Cx43 [79].

Additionally, an indirect influence of Src on GJIC via Src effector kinases has been shown, e.g. for MAPK, PI3K/Akt and PKC signaling pathways [78]. Chronic uncoupling in response to v-Src has been linked to a direct tyrosine phosphorylation of Tyr265 and 247 of Cx43, whereas a serine phosphorylation of Cx43 associated with Src expression does not seem to be essential for long term uncoupling. The acute closure of GJs immediately after Src expression in a “ball and chain” mechanism is not associated with a Src dependent tyrosine phosphorylation, but it is mediated by serine phosphorylation of Cx43 at Ser255, 279, 282 by ERK1/2 [80, 81]. The phosphorylation of Cxs by other tyrosine kinases has been shown for Cx32. Diez et al*.* found a phosphorylation at Tyr243 by the epidermal growth factor receptor tyrosine kinase [82].

### Regulation of HCs by phosphorylation

Despite the large number of kinases that phosphorylate Cxs, only PKA, PKC, MAPK and Akt activity have been found to regulate the HC activity [47, 83–88]. Reported phosphorylation sites of connexins, responsible kinases and the effect on HCs are summarized in Table 2 and for Cx43 shown in Fig. 1.

**Table 2:** Reported phosphorylation sites of connexins, responsible kinases and the effect on hemichannels

Kinases	Connexins	Phosphorylated residues	Reported effects on HCs	References
PKA	Cx35/36	n/a	reduced HC permeability	[89, 90]
AKT (PKB)	Cx43	Ser369, Ser373	increased HC permeability	[85, 88]
	Cx26	n/a	increased HC permeability	[92]
PKC	Cx43	Ser368	reduced HC permeability	[47, 83, 84, 86, 93]
MAPK	Cx43	serines 255, 262, 279, 282	reduced HC permeability	[54, 74, 87]

#### cAMP-dependent protein kinase A (PKA)

A few studies have shown that also neuronal HC activity can be controlled by phosphorylation of Cxs. Mitropoulou and colleagues could demonstrate that HCs formed by Cx35 can be regulated by cAMP/PKA. They injected two different fish Cx35 RNAs (from perch and skate) into Xenopus oocytes to investigate dopamine induced PKA signaling on HC functions. The incubation with cAMP caused a dose-dependent inhibition of voltage-activated HC currents only in the presence of perch but not of skate Cx35 [89]. Amino acid sequence comparison revealed that in skate Cx35 the PKA consensus sequence was absent. The PKA dependent inhibition of HC activity was confirmed by site-directed mutations of the PKA phosphorylation site [89]. DeVries and colleagues also demonstrated that dopamine suppressed HC activity in solitary horizontal cells isolated from catfish retinas using whole-cell voltage clamp [90]. This effect was inhibited by blocking the PKA activity, which also suggests the importance of PKA for dopamine induced inhibition of HC activity [90]. Since PKA also activates the phosphatase PP2A, as discussed before for GJs, the inhibition of HC activity could likely be the effect of Cx dephosphorylation.

#### AKT (PKB)

Recently it has been reported that AKT is capable of regulating HCs, specifically those formed by Cx43 [85]. Previous studies showed that AKT was able to phosphorylate Cx43 in vitro, specifically at Ser369 and Ser373 [91]. Batra and colleagues demonstrated that AKT mediated phosphorylation of HC formed by Cx43 (Cx43-HC) is critical for bone formation and remodeling under mechanical stimulation [85]. They showed that Cx43-HC opening induced by shear stress was dependent on phosphorylation of Cx43 at Ser 369/Ser373 in MLO-Y4 osteocytic cells [85]. Moreover, Cx43 mutated at this site (S369A or S373A) failed to block shear stress induced HC opening [85]. Salas and colleagues showed that metabolic inhibition by blocking the ATP production increased Cx43 HC activity in HeLa cells [88]. They demonstrated that metabolic inhibition induced a rapid and transient activation of AKT that was necessary to increase the number or expression of Cx43-HCs on the cell surface. The inhibition of AKT or the expression of mutated Cx43 (S373A) reduced the activity of Cx43-HCs [88]. However, Figueroa and colleagues demonstrated that linoleic acid increased Cx26-HC opening by activation of PI3K/AKT, because specific PI3K/AKT inhibitors reduced Cx26-HC opening [92]. Whether these HC changes are mediated via phosphorylation of Cx26 is not clear yet [92].

#### Protein kinase C (PKC)

It is well documented that inhibition of PKC increases the Cx43-HC activity [47, 83, 84, 86]. Studies in *Xenopus* oocytes expressing recombinant Cx43 showed that fluorescent dye (5(6)-carboxyfluorescein) uptake via HCs was increased by different blockers of PKC activity (calphostin C, bisindolylmaleimide or chelerythrine) [47]. The amino acid Ser368 has been identified as regulatory site of PKC in Cx43-HC [47, 83, 84]. HCs formed by Cx43 mutated at serine 368 (S368A) were not permeable to sucrose or Lucifer yellow [47, 83, 84]. Permeability assays performed with proteoliposomes loaded with radiolabelled probes revealed that phosphorylation of all Cx43 subunits by PKC inhibited the HC permeability to large but not to small hydrophilic solutes [83]. It also has been demonstrated that chelerythrine, a specific inhibitor of PKC, prevented the inhibitory effect of thrombin on the uptake of lucifer yellow in cultured bovine corneal endothelial cells [93]. Other studies performed in tsA201 cells (a cell line derived from human embryonic kidney cells) that expressed recombinant Cx43, showed that phorbol 12-myristate 13-acetate (PMA) inhibited the macroscopic Cx43-HC conductance [86]. This effect was prevented by bisindolylmaleimide, a specific inhibitor of PKC activity [86]. The treatment with bisindolylmaleimide also increased the total membrane current in Cx43 expressing tsA201 cells [86]. In addition, Hawat and colleagues showed that conventional and novel PKC isoforms are involved in the regulation of Cx43 HCs [86].

#### MAPK

In vitro studies showed that MAPK phosphorylated Ser255, Ser262, Ser279, and Ser282 of Cx43 [54, 74]. Subsequently, functional studies in lipid vesicles containing Cx43-HCs pre-loaded with fluorescent probes (e.g., LY) showed that Cx43-HCs were regulated by MAPK phosphorylation [87]. Kim and colleagues demonstrated that phosphorylation of Cx43 by MAPK reduced the permeability of liposomes containing dephosphorylated Cx43 [87].

## Regulation of GJs and HCs by the redox potential

Changes in the redox potential are probably the broadest control mechanism of ion channels under physiological and pathological conditions [94]. Several ion channels are regulated by one or more oxidant or reducing molecules, including Cxs [95]. Among the large number of oxidant molecules that modulate ion channels, in this review we will focus on three gaseous transmitters so far characterized: nitric oxide (NO), carbon monoxide (CO), hydrogen sulfide (H_2_S) [96], and their effects on GJ channels and HC properties. Regarding the oxidant gas, sulfur dioxide (SO_2_) [97], to our knowledge there are no reports about its role in modulating the permeability of Cx based channels. Reported sites of Cx43 affected by the redox potential are shown in Fig. 1.

### Regulation of GJs by nitric oxide

NO is a gaseous transmitter that is formed by the action of an enzyme family known as nitric oxide synthases (NOS) [98]. There are three isoforms of this enzyme, the endothelial NO synthase (eNOS), mainly expressed in endothelial cells, the neuronal NO synthase (nNOS), largely expressed in nervous tissues, and an inducible one (iNOS), expressed by cells in response to tissue damage [99, 100]. Once produced by the cells, NO can diffuse to neighboring cells and act in two possible ways: i) directly through the S-nitrosylation of cysteine groups [101–103] and ii) indirectly through activation of the guanylyl cyclase, which in turn increases the cGMP levels activating the PKG [103, 104]. In the case of S-nitrosylation, NO effects can be mediated by direct S-nitrosylation of the Cx itself or indirectly by S-nitrosylation of proteins involved in post-translational modifications of the Cx (e.g. kinases or phosphatases) [105]. S-nitrosylation of kinases or phosphatases can affect many signal transduction pathways and often leads to an inhibition of the enzyme activity (e.g., ASK1, JNK, Akt, PKC, IkB, SHP1 and SHP2), but to the contrary kinase activation (e.g., cSrc or glucokinase) has also been described [105]. Several kinases known to phosphorylate Cxs can be regulated via S-nitrosylation, e.g. PKC, MAPK, Cdk5 or Akt [105].

#### Heart

In the heart, Kirca et al. suggested that reduced mitochondrial Cx43 is associated with a change of NOS isoforms leading to reduced NOS activity and - thereby - to reduced S-nitrosylation of several proteins. However, a direct S-nitrosylation of Cx43 by NO was not shown in this study [106], but this effect seems to be important for the endogenous cardioprotection, which is decreased in mice lacking Cx43 [107]. A study of Yang et al*.* demonstrated that the increased risk of arrhythmias in response to increased cardiac oxidative stress is associated with a downregulation of Cx43. Oxidative stress triggered the S-nitrosylation of caveolin 1 (Cav1) by enhanced binding of Cav1 to eNOS, which resulted in the dissociation of S-nitrosylated Cav1 from cSrc/Cav1 complexes and subsequent phosphorylation of cSrc. Activated cSrc finally competed with Cx43 and led to a dislocation of Cx43 from ZO-1 disrupting the GJs and increasing their internalization [108].

#### Endothelial cells

Endothelial cells mainly express Cx37, Cx40 and Cx43 [109]. These cells produce NO via activation of the endothelial NOS (eNOS). NO diffuses via the plasma membranes into the cytoplasm of smooth muscle cells inducing their relaxation. In terms of the effect of NO on GJs formed by these Cxs, it has been shown that NO modified endothelial cell coupling both in vivo and in vitro [110–116]. In particular, NO decreased dye coupling and calcium signal propagation mediated by GJs containing Cx37 in a cGMP independent manner [115, 116]. These results were obtained in a heterologous system (HeLa cells), expressing defined subtypes of Cxs as well as in cultured vascular cells (endothelial cells and co-cultures of endothelial cells and smooth muscle cells) and seem to be important for the modulation of myoendothelial GJs [116]. In agreement with these findings, another group of researchers reported that electrical coupling was no longer reduced by NO in endothelial cells derived from Cx37 knockout mice [110]. Whether NO acts directly or indirectly upon Cx37 is still unknown. Both groups [110, 115] showed that membrane expression of Cx37 was not changed by NO [115] excluding changes in the membrane incorporation as shown for Cx40 [112, 117]. In their study, Hoffmann et al*.* demonstrated that NO in the long term increased the insertion of Cx40 into the cell membrane via activation of cGMP/cAMP dependent kinases [112] therefore leading to an increase of GJ permeability. This effect could be mediated either via a phosphorylation of Cx40 by PKA or indirectly by increasing the incorporation of vesicles with Cx40 containing GJ-HCs into the membrane. NO also led to an increased GJ permeability in vessels highly expressing Cx43, but in contrast to Cx40, NO was probably acting directly on Cx43 via S-nitrosylation of Cys271 within the C-terminus [114].

Because NO is a highly reactive molecule eNOS is expected to be in very close proximity to Cxs present in endothelial cells. This was, in fact, observed for at least Cx37 and Cx40 [118, 119], and both Cxs seemed to be involved in the basal NO production [120]. For a detailed revision of the effect of NO on endothelial cells, see also [121].

#### Brain

By the mid-nineties it was shown in astrocytes that NO produced by iNOS after LPS exposure, decreased GJIC in a peroxynitrite anion (ONOO-) dependent manner [122]. Later on, it could be demonstrated that a downregulation of caveolin-3 was additionally required for this effect [123]. Similarly, in primary cultures of astrocytes from streptozotocin (STZ)-diabetic rats (characterized by increased levels of reactive oxygen-nitrogen species) 75 % of these cells were less coupled compared to non-diabetic astrocytes [124]. Interestingly, the communication between astrocytes was restored by applying dithiothreitol (DTT) to the bath solution [124], which inhibited S-nitrosylation. This suggests that NO induced the S-nitrosylation of Cx43 (probably the major Cx target of diabetes) in astrocytes, which in turn decreased GJIC [124]. However, Retamal and colleagues showed that the intercellular communication was decreased in astrocytes exposed to a conditioned media from microglia exposed to LPS or pro-inflammatory cytokines, which could be restored by inhibition of p38 MAPK but not by DTT [125]. Therefore, NO might exert its effects in different ways and through diverse mechanisms that are not completely understood. However, it remains to be elucidated whether GJs formed by Cx43 in astrocytes can be S-nitrosylated both in vitro and/or in vivo.

In the case of neurons, seminal experiments performed by Rörig and Sutor, demonstrated that a NO/cGMP dependent mechanism decreased GJIC between neurons within the neocortex [126]. In the developing brain, GJs - built by at least Cx26 and Cx36 [127] - were modified by NO/cGMP leading to a reduced permeability of the intercellular channels GJs [126]. In contrast, in neostriatal neurons NO increased gap junctional coupling, which was prevented in the presence of a NO synthase inhibitor [128]. Unfortunately, the mechanism underlying this effect was not analyzed in this study. The production of NO can be stimulated by glutamate that induces a calcium influx necessary for NOS activation. The modulation of GJs by NO/cGMP could therefore be a link between glutamatergic synaptic transmission and gap junctional communication [129]. In HeLa cells transfected with the neuronal Cx35, NO reduced intercellular coupling [59]. Two pathways have been suggested: one acting via the guanylyl cyclase and the other modifying the PKA activity. Nevertheless, both pathways involve changes of the phosphorylation at several serines within the C-terminus of Cx35 [59].

#### Retina

The retina is a tissue that enables the transduction of light into information to be processed by the brain [130]. GJs are essential for the retinal development and its function and, therefore, all retinal cell types express at least one Cx subtype [131]. In response to light, rabbit amacrine cells release dopamine and NO [132]. The activation of the NO/cGMP/PKG dependent pathway, induced the inhibition of GJIC of horizontal cells of turtle [133], rabbit [134], goldfish [135] and bass [136], suggesting that both NO and GJIC are involved in the light adaptation. Similarly, GJIC between AII amacrine and bipolar cells was moderately decreased after SNAP and 8-Br-cGMP exposition [137]. Therefore, NO modulates the light processing in the retina through regulation of the intercellular communication mediated by GJs [137].

### Regulation of HCs by nitric oxide

HCs participate in several physiological and pathological processes [138]. For that reason, they are tightly controlled by several mechanisms, including the redox potential [95]. In this context, NO appears to be a very important molecule that rules HC activity [95]. Thus, in cultured astrocytes, exogenous application of the physiological NO-donor GSNO was associated with an increase in Cx43-HC activity probably mediated by S-nitrosylation of Cx43 [139]. Furthermore, activated microglia can open Cx43-HCs in astrocytes in a NO-dependent manner since this effect was completely blocked by the NOS blocker L-NAME and also by DTT [125]. Interestingly, DTT is also able to increase HC activity in both astrocytes and HeLa cells [140]. These opposing effects of DTT might be explained by the dependency from the metabolic cell status. DTT increased the HC activity under control conditions, but decreased the HC activity after at least 40 min of metabolic inhibition [140], indicating that NO effects are more complex possibly involving changes in phosphorylation and/or S-nitrosylation of Cx43 [140]. It has been proposed that S-nitrosylation in Cx43 HCs could occur at Cys271 as a regulatory site in Cx43 GJs [114], therefore, it is possible that this residue is involved in Cx43-HC regulation induced by NO. Experiments with Cx46 showed that NO is able to modulate the voltage sensitivity, opening/closing kinetics and permeability of Cx46 HCs [141]. The effect of NO on HCs seems to be dependent on the Cx type, for example HCs formed by Cx37, Cx40 and Cx43 can be opened by NO whereas Cx32 HCs were closed by NO [142].

Not only Cxs but also pannexin channels can be regulated via NO. An inhibitory effect of NO in a cGMP/PKG dependent way was demonstrated by Poornima et al*.* [143]. They showed that NO donors reduced Panx1 currents in HEK293 cells and that inhibition of the soluble guanylate cyclase or of PKG blocked the NO effect [143].

## Regulation of GJs and HCs by carbon monoxide

CO is a toxic molecule because it binds with high affinity to hemoglobin [144, 145]. However, there is a growing body of evidence showing that CO can be involved as a physiological molecule in several cellular processes [146]. Thus, CO is produced by heme oxygenase enzymes under physiological conditions by decomposition of heme to biliverdin [147]. Recently, it has been demonstrated that CO can act as a novel HC modulator [148, 149]. In a study from León-Paravic et al*.* the application of CO donors (CORM-A1, CORM-2 and CORM-3) to the bath solution inhibited Cx46 HC currents in *Xenopus laevis* oocytes [148]. This effect was fully prevented by the addition of the CO scavenger hemoglobin and correlated with Cx46 carbonylation, which in turn, produced important protein structural rearrangements. Additionally, Cx43-HCs in HeLa cells were also inhibited by CO donors. The HC inhibition observed had an IC_50_ of about 3.4 μM (the physiological concentration is a few micromolar), making Cx46- and Cx43-HCs to excellent sensors of physiological and pathological changes in CO concentration [148]. This study further demonstrated that Cx46-HCs lacking extracellular-loop cysteines were much less sensitive to CO and that CO induced HC inhibition was fully recovered by addition of reducing agents to the bath solution (e.g. GSH or DTT). This suggests that extracellular-loop cysteines are important for CO induced Cx46-HC inhibition [148]. So far, there are no additional reports demonstrating a CO effect on other Cxs and there is no evidence on CO induced effects upon GJ channels. However, due to the inhibitory effect of CO on HCs it might be possible that CO can reduce GJIC probably due to reduced GJ formation rather than a direct action.

## Regulation of GJs and HCs by hydrogen sulfide

Three enzymes are responsible for the formation of the gaseous transmitter H_2_S from L-cysteine: cystathionine γ-lyase (CSE), cystathionine β-synthase (CBS), and 3-mercaptopyruvate sulfurtransferase (3-MST) [150]. Sulfhydration occurs when H_2_S reacts with cysteine residues to form SSH or persulfide groups [151]. The modification of Cx HC properties by H_2_S has not been reported yet. However, it has been shown that H_2_S improved the expression of Cx43 in cardiomyocytes [152], which improves the cardiac outcome, decreases hypertrophy, and reduces fibrosis. Additionally, application of H_2_S decreases platelet aggregation probably by interfering with GJ channels, at least in part [153].

## Conclusions

Changes of environmental conditions, e.g. the presence of growth factors, cytokines, wounding, ischemic conditions, changes in the redox potential, but also of cellular processes as cell cycle progression often lead to post-translational modifications of Cxs. Thus, to respond to acute changes of environmental or cellular conditions cells have developed an appropriate mechanism to regulate their intercellular communication with neighboring cells or their communication with the extracellular space via HCs. The great amount of excellent studies and reports clearly demonstrate the importance of post-translational changes of Cxs for the regulation of HCs or intact GJ channels. In the case of phosphorylation, similar roles regarding the channel permeability have been described for different Cx types, but there are also some exceptions, too. The effect on channel permeability of HCs is consistent with that of GJs after phosphorylation of Cxs. However, the effect of NO on GJ channels or on HCs is not unitary. Therefore, it is not possible to establish a common pattern of action of NO on Cx HCs or GJ channels. Based on these reports it has been shown that not only the Cx type is important, but also the different intracellular pathways activated by NO in different types of cells. The complex situation of a certain cell type and its environment is critical for the net effect of post-translational modifications and will also depend on the metabolic cell status and kinase/phosphatase expression under a given condition. The abundance of literature demonstrate that post-translational modifications of Cxs are of great importance in modifying HC activity or GJIC and in future probably additional sites for post-translational modifications will be identified.
